# Food allergy and anaphylaxis – 2051. Economic burden of anaphylaxis in the United States

**DOI:** 10.1186/1939-4551-6-S1-P134

**Published:** 2013-04-23

**Authors:** Ray Wolf, David Alexander Sclar, Mary-Ellen Marx

**Affiliations:** 1 Medical Affairs, Mylan Specialty L.P., Basking Ridge, NJ, USA; 2 Department of Health Policy & Administration, Washington State University, Spokane, WA, USA

## Background

Although the prevalence of allergy and incidence of anaphylaxis are rising globally, there exists a paucity of data as to the economic burden of anaphylaxis. This inquiry reports the estimated magnitude of national fiscal outlays (direct and indirect) in the United States (US), and intensity of demand by health service area.

## Methods

We employed Monte-Carlo simulation, a decision-analytical framework parameterized by stochastic and deterministic components, to yield a national (US) burden of illness profile for anaphylaxis. Findings are based on the US population in 2010, and are reported in 2010 dollars (US). The prevalence of the at-risk population by type of exposure (food; insects; medication; latex), resulting use of health services (direct costs), and death, were discerned from national survey data from the US National Center for Health Statistics, and the medical literature. Indirect costs included lost productivity (earnings), for both patients and caregivers, and mortality. The methodology used in this study is applicable on an international basis.

## Results

In 2010, the estimated US population at risk for anaphylaxis ranged between 3.7 and 48.7 million (median = 14.4 million). The estimated incidence of anaphylaxis ranged between 50,446 and 657,330 (median = 211,874). Direct expenditures ranged between $288 million and $3.7 billion (median = $1.2 billion). Indirect expenditures ranged between $145 million and $1.9 billion (median = $609 million). The point-estimate for direct expenditures for epinephrine was $294 million. Net of the point-estimate for direct expenditures of epinephrine, and accounting for biphasic anaphylaxis, both direct and indirect expenditures far exceeded the expenditure of equipping at-risk patients with epinephrine autoinjectors.

## Conclusions

The extent of under-diagnosis and under-reporting of anaphylaxis in the US precludes an exacting measure of the burden of illness. Our results suggest the burden of illness due to anaphylaxis in the US is far greater than previously reported. Similar results may apply internationally.

